# Prospects for Universal Influenza Virus Vaccine

**DOI:** 10.3201/eid1204.051020

**Published:** 2006-04

**Authors:** Walter Gerhard, Krystyna Mozdzanowska, Darya Zharikova

**Affiliations:** * The Wistar Institute, Philadelphia, Pennsylvania, USA

**Keywords:** influenza virus, universal vaccine, antibody, T cell

## Abstract

The current vaccination strategy against influenza A and B viruses is vulnerable to the unanticipated emergence of epidemic strains that are poorly matched by the vaccine. A vaccine that is less sensitive to the antigenic evolution of the virus would be a major improvement. The general feasibility of this goal is supported by studies in animal models that show that immunologic activities directed against relatively invariant viral determinants can reduce illness and death. The most promising approaches are based on antibodies specific for the relatively conserved ectodomain of matrix protein 2 and the intersubunit region of hemagglutinin. However, additional conserved determinants for protective antibodies are likely to exist, and their identification should be encouraged. Most importantly, infection and current vaccines do not appear to effectively induce these antibodies in humans. This finding provides a powerful rationale for testing the protective activity of these relatively conserved viral components in humans.

Current influenza virus vaccines attempt to induce strong antibody responses against the viral glycoproteins hemagglutinin (HA) and, with lesser emphasis, neuraminidase (NA) because their protective efficacy is well documented. Thus, typical HA-specific antibodies neutralize viral infectivity and fully protect against infection when they are present at sufficient concentration in the lining fluid of the respiratory tract, and typical NA-specific antibodies inhibit the release of newly formed virus from infected host cells and thus limit the spread and shedding of virus during infection. Current vaccines are highly effective in children and adults (70%–90%), although not in those >65 years of age (30%–50%) (1). Apart from their limited efficacy in the elderly, a major drawback of current vaccines is that the principal vaccine targets, most notably the distal region of HA, are subject to continuous alteration in circulating epidemic virus strains (2,3). This process, termed antigenic drift, results from the high mutation rate of the viral genome and the continuous selection of mutants with improved replication characteristics in the immune human host population. On average, the prevalent influenza A virus strain acquires 3–4 amino acid changes per year in HA, with most being located in the regions recognized by protective antibodies. Every 2 to 5 years, the accumulation of mutations results in a major antigenic drift away from the previously circulating strains (4). A more drastic antigenic change, termed antigenic shift, occurs if a new HA subtype is introduced into the pool of human virus strains by reassortment of genes between animal and human strains or by direct transmission of strains from an animal reservoir to humans, as has occurred recently with strains of H5N1, H7N7, and H9N2 (1). Accordingly, the influenza vaccine must be updated on a regular basis to reflect the antigenic changes that occur in the pool of circulating virus strains. Because vaccines have to be manufactured before the actual epidemic strains are known, a failure to anticipate emergence of a strain with major antigenic drift or shift relative to the vaccine will result in a substantial reduction or abrogation of vaccine-mediated protection.

While antibodies to the immunodominant, but highly variable, regions of HA and NA can provide potent virus strain–specific protection, the existence of weaker and more broadly protective immune activities directed to less variable regions of viral proteins has long been known (5). These protective activities have collectively been termed heterotypic or heterosubtypic immunity because they provide a measure of protection against viruses of distinct subtypes. Because of their potential for broadening vaccine-mediated protection in humans, they have been studied extensively in animals and found to be mediated predominantly by virus-specific memory T cells (6,7), antibodies (8–10), or a combination of both (11–13). The reason for these differences in the relative strength of T-cell and antibody-mediated protection is not clear but could be attributable to differences in vaccination procedures, virus challenge, and read out (how protection was measured) between the various studies. Pros and cons of some of these activities in terms of their potential for development of a broadly protective, "universal" influenza vaccine are briefly discussed below.

## Memory T Cells

A large fraction of the virus-specific T-cell response in mice and humans is directed to conserved determinants of viral core proteins, and many studies in mice have shown that memory T cells can accelerate recovery and reduce illness on virus challenge. Cytotoxic T (Tc) cells were found to be more protective than helper T cells, and among Tc cells, protective activity was shown to depend on their frequency (number of virus-specific cells/total cells), cytokine secretion profile, memory type (central vs. effector), and even fine specificity. However, in contrast to findings in mice, the protective value of memory Tc cells in humans remains controversial. The classic study by McMichael et al. (14) indicated that presence of memory Tc cells in blood, which could give rise to Tc cells on stimulation in vitro, correlated with reduced virus shedding 3–4 days after volunteers were challenged with a wild-type virus, but had no significant effect on illness. Subsequent studies performed in children found no significant difference in shedding of attenuated vaccine strains in patients who had recovered from previous infection with a vaccine or natural strain of a different subtype than did study participants who had no evidence of previous virus exposure (15,16). Similarly, children vaccinated with an H1N1 strain showed no difference in attack rate and febrile respiratory illness during exposure to natural epidemic H3N2 virus from controls who received a placebo (17).

Although the presence of memory Tc cells in the vaccinated children was not demonstrated experimentally, it can be implied based on findings that infection with a live, attenuated vaccine or natural virus strain typically stimulates a Tc-cell response in humans. Taken together with the observation that the degree of antigenic change (drift, shift) is a major determinant of epidemic severity, little evidence exists for a substantial protective role of subtype cross-reactive memory Tc cells in human influenza virus infection; the contribution of Tc cells per se in the control of the infection is not questioned, only whether memory Tc cells provide a further improvement.

Vaccine-induced or natural upper respiratory tract infection in humans may not engender an optimally protective memory Tc-cell population because of insufficient number or composition. However, a large number of memory T cells may also result in immunopathologic manifestations (14,18), which tend to be associated with excessive inflammatory responses in acute infections. Thus, a universal vaccine based on the induction of a strong memory-Tc response might necessitate a difficult balancing act between protection and immunopathologic changes. Unless one can identify a particularly protective memory Tc-cell population that is poorly induced by natural or vaccine-induced infection, the nondiscriminatory enhancement of memory T-cell populations may not be a promising approach for a universal influenza vaccine.

## Antibodies Specific for Conserved Viral Determinants

A precondition for antibody-mediated protection is the accessibility of the viral antigen to antibody on infectious virus particles, intact infected cells, or both. This accessibility restricts the potential targets to conserved structures of the ectodomains of viral transmembrane proteins HA, NA, and M2, in the case of influenza A viruses, and HA, NA, NB, and BM2, in the case of influenza B viruses. Results of studies reported thus far have focused on M2 of influenza A and HA of influenza A and B viruses.

### M2 of Influenza A Viruses

M2 forms tetramers that exhibit pH-inducible proton transport activity. It regulates the pH of the viral core after virus uptake into the host cell's endosomal compartment during initiation of infection and subsequently of vesicles that transport the viral transmembrane proteins to the cell surface during the late stage of infection. M2 tetramers are expressed at high density in the plasma membrane of infected cells and are well accessible to M2e-specific antibodies in this location, but only a few copies become incorporated into the envelope of mature infectious virus particles (19,20). M2 has a small, nonglycosylated ectodomain (M2e) of 23 amino acids (aa), not counting the posttranslationally removed N-terminal Met. This region has shown only limited variation among human influenza A viruses. This remarkable degree of structural conservation of M2e is attributable mainly to its genetic relation with matrix protein 1 (M1), the most conserved protein of influenza A viruses with which it shares coding sequences. Thus, aa residues 1–9 of M2e and M1 are encoded by the same nucleotides in the same reading frame and aa 10–23 of M2e and 239–252 of M1 in a different reading frame.

Studies by several groups conducted in mice and ferrets have shown that M2e-specific antibodies, while they did not prevent infection, restricted subsequent virus replication and reduced illness and proportion of deaths (20–24). This antibody response was only poorly induced by infection, both in mice (22) and humans (24,25). A likely reason for the poor M2e-specific antibody response is extensive antigenic competition with HA- and NA-specific responses (26). Thus, in view of the >10-fold difference in ectodomain size, the frequency of M2e-specific precursor B cells must be orders of magnitude lower than the frequencies of HA- and NA-specific precursor B cells. Assuming that most immunogenic entities generated in the course of infection contain a mixture of all 3 transmembrane proteins, most M2e may be taken up by HA- and NA-specific B cells, leaving little or none for B-cell receptor–mediated uptake and processing by M2e-specific precursor B cells. Note that the same phenomenon results also in a suppression of the NA-specific antibody response by immunodominant HA-specific B cells (26). Such competition can be avoided by presenting individual antigens on physically distinct immunogenic entities to the immune system (27). The substantial M2e-specific antibody responses seen in mice after vaccination with dedicated M2e vaccines (20–24) supports the above explanation.

In view of the poor or absent M2e-specific antibody response in humans, confirming the genetic stability of M2e was essential when the virus was propagated in an immune environment. Replication of A/PR/8/34(H1N1) (PR8) virus for >3 weeks in severe combined immunodeficient (SCID) mice that were chronically treated with M2e-specific monoclonal antibodies (MAbs) resulted in the emergence of M2e-escape mutants (28). However, only 2 distinct escape mutants emerged, 1 with a replacement of Pro at position 10 by Leu (P10L) and the other with a replacement of the same Pro by His (P10H) (28). Each of these mutants was isolated repetitively from many distinct mice treated with distinct M2e-specific MAbs, which indicates that they represented essentially the entire range of escape mutants capable of arising from the PR8 wild-type virus under the given experimental conditions. No escape mutants emerged after 11 consecutive passages of PR8 in BALB/c mice that had been actively vaccinated with M2e (unpub. data). In addition, incorporating determinants of potential escape mutants into a polyvalent universal M2e vaccine would likely further impede emergence of escape mutants. Indeed, preliminary studies have shown that no escape mutants emerged in SCID mice treated with a combination of MAbs specific for M2e of wild-type PR8 and the P10H and P10L escape mutants (unpub. data). Thus, although M2e is not totally invariant, it is remarkably stable, even under immune pressure.

Several vaccination strategies have been evaluated in mouse and ferret models, including M2-expressing recombinant viruses, M2 recombinant proteins (20,21), M2-encoding plasmid DNA (29), and synthetic M2e peptides that were chemically linked to carrier proteins or synthetically linked to defined helper T-cell determinants (22–24). In most studies in which induction of an antibody response was confirmed, M2e-specific immunity reduced illness, but did not entirely prevent it. The best protection was reported for mice vaccinated by the intranasal route with an M2e-hepatitis B core fusion protein construct and detoxified heat-labile *Escherichia coli* enterotoxin adjuvant; almost none of these mice died after a virus challenge that killed 90% of control mice (21). However, in contrast to the significant protection seen in most mouse models, pigs vaccinated with recombinant M2e-hepatitis B core protein or plasmid DNA encoding an M2e-nucleoprotein fusion protein showed no protection or even had higher death rates, respectively, after virus challenge (29). This finding needs to be confirmed, and the explanation for it remains unknown. At this time, it serves as a reminder that immune phenomena are complex and that observations made in 1 species may not apply to another. By the same token, good protection in an animal model does not guarantee protection in humans.

Taken together, the observations that M2e shows minimal antigenic variability, even under antibody-mediated pressure in vivo, that M2e-specific antibodies typically restrict virus replication in vivo, and that humans exhibit low or undetectable M2e-specific antibody titers provide a strong rationale for further exploration of an M2e-based vaccine.

### HA of Influenza A and B Viruses

The HA molecule has a large ectodomain of ≈500 aa. A posttranslational cleavage by host-derived enzymes generates 2 polypeptides that remain linked by a disulfide bond. The larger N-terminal fragment (HA1, 320–330 aa) forms a membrane-distal globular domain that contains the receptor-binding site and most determinants recognized by virus-neutralizing antibodies. The smaller C-terminal portion (HA2, ≈180 aa, excluding transmembrane and cytoplasmic domain) forms a stemlike structure that anchors the globular domain to the cellular or viral membrane. Sixteen HA subtypes have been identified among influenza A viruses (30); 3 of these (H1, H2, H3) have been associated with classic influenza isolates, and 3 (H5, H7, H9) have been associated with recent sporadic human isolates (1). Influenza B viruses possess only 1 HA subtype.

Although the degree of sequence diversity between subtypes is great, particularly in the HA1 polypeptides (34%–59% homology between subtypes), more conserved regions are found in HA2 (51%–80% homology between subtypes). The most notable region of conservation is the sequence around the cleavage site, particularly the HA2 N-terminal 11 aa, termed fusion peptide, which is conserved among all influenza A subtypes and differs only by 2 conservative aa replacements in influenza B virus. Part of this region is exposed as a surface loop in the HA precursor molecule (HA0) (31). However, when HA0 is cleaved into HA1/HA2, the newly generated terminals separate, and the hydrophobic fusion peptide becomes tucked into a cavity of the stem (31). As most HA subtypes are cleaved by extracellular enzymes, this surface loop may be accessible to antibody, at least temporarily, on HA0 expressed in the plasma membrane of infected host cells. The protective potential of antibodies directed to this region of HA0 has been explored in 2 studies by immunization of mice with synthetic peptides spanning the cleavage site (32,33). Both studies found that mice vaccinated with a peptide spanning the HA1/HA2 joining region exhibited less illness and fewer deaths on virus challenge (32,33). Most importantly, HA1/HA2 joint-specific antibodies were undetectable in virus-immune human sera (33). These findings make the HA1/HA2 joining region another promising candidate for inclusion in a universal vaccine. Indeed, the authors of 1 study, some of whom had been involved in an M2e-vaccine study, commented that joint-specific immunity in the mouse model was more robust than M2e-specific immunity (33).

Although the HA1/HA2-joining region is the most broadly conserved HA sequence, other determinants on HA2 are shared between a restricted number of subtypes. For instance, a MAb that reduced illness and death in passively immunized mice against viruses of the H1, H2, and H5 subtypes has been described (34,35). This MAb was shown to recognize a conformational epitope of HA2 (36), but no immunogen that could selectively induce this response has been described. A search for determinants shared by a more restricted number of closely related subtypes such as H2 and H5, which display 85% sequence homology in HA2, or shared by members of the same subtype, which typically display >95% sequence homology in HA2 (30), would be worthwhile, particularly since the HA2-specific antibody response appears to be induced less effectively than the HA1-specific response by infection in humans (37). That many HA2-specific antibodies do not display substantial antiviral activities in vitro does not preclude protective activity in vivo because the mere binding of antibody to native HA expressed on infected cells and infectious virus could mediate protective activity by targeting Fc-receptor expressing cells or complement deposition to these structures.

### Other Viral Transmembrane Proteins

To our knowledge, conserved determinants for protective antibodies have not been described for any of the other transmembrane proteins of influenza A and B virus. BM2 of influenza B virus, the homolog of M2, has only a 6-aa-long ectodomain (38). This ectodomain is most likely too small for formation of a BM2-specific epitope because protein epitopes have usually been found to comprise 12–17 contact residues. NB of influenza B virus also shows structural similarities with M2 of influenza A virus, including ion channel activity (39), and has an 18-aa-long ectodomain. However, NB2 has 2 attached carbohydrate chains that can be expected to mask the protein core from recognition by antibody. NA, however, is a good and not sufficiently explored target for cross-protective antibodies. Like HA, it displays a large ectodomain of ≈420 aa. Nine subtypes are recognized among influenza A viruses, while influenza B virus contains 1 subtype. The C-terminal of the polypeptide (≈380 aa) forms a globular head that is anchored to the viral membrane by a flexible stalk. The globular domain contains the enzyme-active site and all known antigenic sites.

Although no cross-protective NA-specific antibody population has been identified, indirect evidence supports the existence of cross-reactive determinants on N1 and N2, the subtypes found in classic human strains. Thus, mice vaccinated first with a mixture of purified N1 and N2 proteins and subsequently boosted with the individual antigens showed a small memory response also against the heterologous subtype (40). Given the ample expression and accessibility of NA on infectious virus and infected host cells, a search for determinants shared between or within subtypes would be worthwhile.

## Conclusions

Studies in animal models have yielded clear evidence for the existence of antibody populations that are directed to relatively invariant determinants of the ectodomains of viral transmembrane proteins and are capable of substantially reducing, in some cases even preventing, clinical illness resulting from influenza virus infection. Additional highly conserved determinants likely exist, particularly on HA2 and NA polypeptides, which can serve as targets for protective antibodies. These targets should be identified for 2 reasons. First, with the exception of the fusion peptide, none of the presently identified "conserved" determinants is totally invariant, and each of these relatively invariant determinants may show increased variability under specific immune pressure. Second, incorporation of several conserved targets in a universal vaccine may decrease the likelihood and rate of emergence of escape mutants and increase the strength of protection.

None of the identified broadly protective antibody populations has been found consistently and at appropriate concentrations in human sera, which indicates that neither is effectively induced by natural infection or current vaccines. Therefore, the observation that heterosubtypic protection in humans tends to be low does not exclude the possibility of substantial protection by these antibody populations in humans if it can be induced by a specific vaccine. A focused search will likely show additional relatively conserved target structures for protective antibodies. Any of these responses, if not already induced effectively by infection or current vaccines, will be worth pursuing for incorporation into a universal vaccine. The main difficulty may be to develop in each case an immunogenic moiety that can effectively induce the desired antibody population. However, even if an appropriate vaccine for induction of a desired broadly protective antibody response cannot be developed, through this research, humanized antibody reagents may be generated that can be used to treat life-threatening human infections. In view of the potential rewards, the universal vaccine approach should be further explored in animal models and its protective efficacy assessed in humans.

None of the universal vaccines studied thus far in animal models has achieved the level of protection provided with current vaccines. Still, an optimized polyvalent universal vaccine, while not preventing infection, may prevent clinical disease, as has been reported already for 2 vaccination modalities (21,23). If the same results applied to humans, a universal vaccine might replace the current vaccine. Alternatively, if a universal vaccine can only reduce, but not prevent, clinical disease in humans, it could still be used as adjunct to current vaccines and provide increased resistance in case of the unanticipated emergence of a major drift variant or new subtype. Newborns, who are at risk for severe disease, would then receive at least some protection by maternal antibodies. In the elderly, another high-risk population, a universal vaccine may be particularly advantageous because the protective antibodies are generated by memory B cells that tend to be maintained into old age and can be recalled by booster vaccination. In contrast, the efficacy of current inactivated vaccines depends greatly on the ability to mount a strong response to novel (strain-specific) determinants generated through antigenic drift and shift on HA and NA. This response requires naive B cells, whose frequency tends to decrease with increasing age. When all factors are taken into account, protection against influenza virus infection likely can be improved by a universal vaccine.
